# Supplementary material for: Accurate and interpretable drug-drug interaction prediction enabled by knowledge subgraph learning
Source: Commun Med (Lond). 2024 Mar 28;4:59. doi: 10.1038/s43856-024-00486-y (PMC10978847; doi:10.1038/s43856-024-00486-y)
Supplement: Supplementary file 2 — Supplementary Information [file 43856_2024_486_MOESM2_ESM.pdf]

Supplementary Table 1: Comparing the proposed KnowDDI with existing works.

Method	External KG	Drug-pair-aware Representation	Adding New Edges	Removing Irrelevant Edges	Interpretable Paths
GAT [44]	✗	✗	✗	✗	✗
Decagon [10]	✗	✗	✗	✗	✗
SkipGNN [11]	✗	✗	✗	✗	✗
GraIL [22]	✓	✗	✗	✗	✗
KGNN [21]	✓	✗	✗	✗	✗
DDKG [50]	✓	✗	✗	✗	✗
SumGNN [23]	✓	✓	✗	✓	✓
LaGAT [24]	✓	✓	✗	✗	✓
KnowDDI	✓	✓	✓	✓	✓

Supplementary Note 1: Complete algorithm of KnowDDI

We first provide the procedure of extracting drug-flow subgraph $\bar{S}_{h,t}$ for drug-pair (h, t) in Algorithm 1. Then, we present the training and testing procedures of KnowDDI in Algorithm 2 and Algorithm 3 respectively.

Algorithm 1 Drug-flow Subgraph Extraction: DSextract($\mathcal{G}, h, t, K, P$).

Require: a combined network $\mathcal{G} = \{\mathcal{V}, \mathcal{E}, \mathcal{R}\}$ which combines the DDI graph and an external KG, target drug-pair (h, t), hyperparameters K, P;

$\mathcal{V}_h^{(0)} = \{h\}, \mathcal{V}_{h\to t}^{(P)} = \{t\}$; ▷ initialize node sets
$\mathcal{N}_K(h) = \{s | d(s, h) \leq K, s \in \mathcal{V}\}$; ▷ extract K-hop (undirected) neighborhood of h
$\mathcal{N}_K(t) = \{s | d(s, t) \leq K, s \in \mathcal{V}\}$; ▷ extract K-hop (undirected) neighborhood of t
$\bar{\mathcal{E}}_{h,t} = \{(u, r, v) | u, v \in \mathcal{N}_K(h) \cap \mathcal{N}_K(t), (u, r, v) \in \mathcal{E}\}$; ▷ extract facts in the interaction of $\mathcal{N}_K(h)$ and $\mathcal{N}_K(t)$
$\hat{\mathcal{E}}_{h,t} = \bar{\mathcal{E}}_{h,t} \cup \{(t, r_{\text{identity}}, t)\}$; ▷ add identity relation r_{identity} for t such that all relation paths between h and t will have length P
for $p = 1, 2, \ldots, P$ **do**
  $\mathcal{E}_h^{(p)} = \{(u, r, v) \in \hat{\mathcal{E}}_{h,t} | u \in \mathcal{V}_h^{(p-1)}\}$; ▷ record the p-hop outgoing edges of h
  $\mathcal{V}_h^{(p)} = \{v | (u, r, v) \in \mathcal{E}_h^{(p)}\}$; ▷ record tail entity nodes of edges in $\hat{\mathcal{E}}_h^{(p)}$
end for
if $\mathcal{V}_h^{(P)} \cup \mathcal{V}_{h\to t}^{(P)} = \emptyset$ **return** $\mathcal{S}_{h,t} = \{\{h, t\}, \emptyset, \emptyset\}$;
for $p = P, P-1 \ldots, 1$ **do**
  $\mathcal{E}_{h\to t}^{(p)} = \{(u, r, v) \in \mathcal{E}_h^{(p)} | v \in \mathcal{V}_{h\to t}^{(p)}\}$; ▷ record the p-hop incoming edges of t
  $\mathcal{V}_{h\to t}^{(p-1)} = \{u | (u, r, v) \in \mathcal{E}_{h\to t}^{(p)}\}$; ▷ record head entity nodes of edges in $\mathcal{E}_{h\to t}^{(p)}$
  $\mathcal{R}_{h\to t}^{(p-1)} = \{r | (u, r, v) \in \mathcal{E}_{h\to t}^{(p)}\}$; ▷ record relation types of edges in $\mathcal{E}_{h\to t}^{(p)}$
end for
$\bar{\mathcal{V}}_{h,t} = \mathcal{V}_{h\to t}^{(1)} \cup \mathcal{V}_{h\to t}^{(2)} \cup \cdots \cup \mathcal{V}_{h\to t}^{(P)} \cup \{h, t\}$; ▷ obtain the set of nodes in $\mathcal{S}_{h,t}$
$\bar{\mathcal{R}}_{h,t} = \mathcal{R}_{h\to t}^{(1)} \cup \mathcal{R}_{h\to t}^{(2)} \cup \cdots \cup \mathcal{R}_{h\to t}^{(P)}$; ▷ obtain the set of relations in $\mathcal{S}_{h,t}$
$\bar{\mathcal{E}}_{h,t} = \mathcal{E}_{h\to t}^{(1)} \cup \mathcal{E}_{h\to t}^{(2)} \cup \cdots \cup \mathcal{E}_{h\to t}^{(P)}$; ▷ obtain the set of facts in $\mathcal{S}_{h,t}$
return $\bar{\mathcal{S}}_{h,t} = \{\bar{\mathcal{V}}_{h,t}, \bar{\mathcal{E}}_{h,t}, \bar{\mathcal{R}}_{h,t}\}$ specific to (h, t).

Algorithm 2 Training procedure of KnowDDI.

Require: a DDI graph $\mathcal{G}_{\text{DDI}} = \{\mathcal{V}_{\text{DDI}}, \mathcal{E}_{\text{DDI}}, \mathcal{R}_{\text{DDI}}\}$ which contains the known DDI fact triplets, an external KG $\mathcal{G}_{\text{KG}} = \{\mathcal{V}_{\text{KG}}, \mathcal{E}_{\text{KG}}, \mathcal{R}_{\text{KG}}\}$ which carries abundant knowledge in biomedicine and healthcare, and hyperparameters L, T, P, α, γ.

```
initialize θ_g, θ_k randomly;
merge G_DDI and G_KG into the large combined network G = {V, E, R};
initialize e_v^(0) for v ∈ V by random Gaussian or pretrained KG embeddings;
while not done do
    for l = 1, ..., L do
        obtain generic embedding e_v^(l) by equation (1) and equation (2);
    end for
    for all (h, r, t) ∈ E_DDI do
        extract its drug-flow subgraph S̄_{h,t} by DSextract(G, h, t, K, P) (Algorithm 1);
        initialize node embedding h_v^(0) as e_v^(L) and graph structure A_{h,t}^(0) as Ā_{h,t};
        for τ = 1, ..., T do
            estimate graph structure A_{h,t}^(τ) by equation (6) and equation (7);
            refine node embedding h_v^(τ) by equation (10);
        end for
        obtain knowledge subgraph S_{h,t} corresponding to graph structure recorded in A_{h,t}^(T);
        obtain subgraph embedding h_{S_{h,t}} of S_{h,t} by equation (11);
        obtain class prediction ŷ_{h,t} by equation (12);
    end for
    if multiclass DDI prediction then optimize θ_g and θ_k w.r.t. equation (13).
    else if multilabel DDI prediction then optimize θ_g and θ_k w.r.t. equation (14).
    end if
end while
return optimized θ_g and θ_k.
```

Algorithm 3 Testing procedure of KnowDDI.

Require: a testing drug-pair (h', t'), a trained KnowDDI with optimized $\boldsymbol{\theta}_g$ and $\boldsymbol{\theta}_k$, a combined network $\mathcal{G} = \{\mathcal{V}, \mathcal{E}, \mathcal{R}\}$ which combines the DDI graph and external KG, and hyperparameters L, T, P, α, γ.

initialize $\mathbf{e}_v^{(0)}$ for $v \in \mathcal{V}$ by random Gaussian or pretrained KG embeddings;
for $l = 1, \dots, L$ **do**
    obtain generic embedding $\mathbf{e}_v^{(l)}$ by equation (1) and equation (2);
end for
extract its drug-flow subgraph $\bar{\mathcal{S}}_{h',t'}$ by DSextract($\mathcal{G}, h', t', K, P$) (Algorithm 1);
initialize node embedding $\mathbf{h}_v^{(0)}$ as $\mathbf{e}_v^{(L)}$ and graph structure $\mathbf{A}_{h',t'}^{(0)}$ as $\bar{\mathbf{A}}_{h',t'}$;
for $\tau = 1, \dots, T$ **do**
    estimate graph structure $\mathbf{A}_{h',t'}^{(\tau)}$ by equation (6) and equation (7);
    refine node embedding $\mathbf{h}_v^{(\tau)}$ by equation (10);
end for
obtain knowledge subgraph $\mathcal{S}_{h',t'}$ corresponding to graph structure recorded in $\mathbf{A}_{h',t'}^{(T)}$;
obtain subgraph embedding $\mathbf{h}_{\mathcal{S}_{h',t'}}$ of $\mathcal{S}_{h',t'}$ by equation (11);
obtain class prediction $\hat{\mathbf{y}}_{h',t'}$ by equation (12);
if multiclass DDI prediction **then** return $\arg\max_i [\hat{\mathbf{y}}_{h',t'}]_i$ which is the relation with the highest possibility,
else if multilabel DDI prediction **then** return $\hat{\mathbf{y}}_{h',t'}$.
end if

Supplementary Fig. 1: **Different knowledge subgraph generation strategies for KnowDDI on Drug-Bank.**

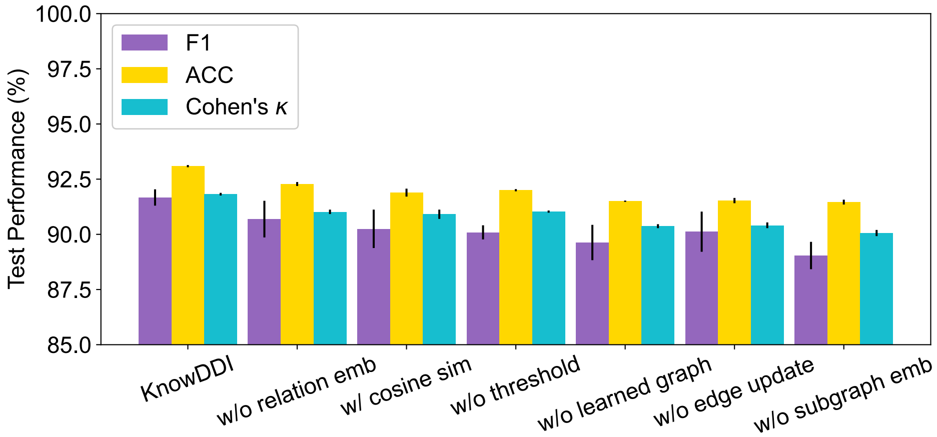

This bar plot illustrates the test performance (%), with each bar's height representing the mean result and the error bars indicating the standard deviation, both derived from five independent runs (n=5).

Supplementary Note 2: Different knowledge subgraph generation strategies

We compare the KnowDDI with variants changing the knowledge subgraph generation strategy of KnowDDI: (i) **w/o relation emb**: remove relation embedding $\mathbf{h}_r$ in equation (6); (ii) **w/ cosine sim**: replace equation (6) by cosine similarity between $\mathbf{h}_u^{\tau-1}$ and $\mathbf{h}_v^{\tau-1}$; (iii) **w/o threshold**: remove threshold γ in equation (7); (iv) **w/o learned graph**: use the graph structure $\mathbf{A}_{h,t}^{(0)}$ of drug-flow subgraph directly, which can be achieved by always setting $\mathbf{A}_{h,t}^{(\tau)} = \mathbf{A}_{h,t}^{(0)}$ in equation (10); (v) **w/o edge update**: calculate $\mathbf{C}_{h,t}^{(\tau)}$ in equation (6) using generic embedding $\mathbf{h}_u^{(0)}$ for all layers; and (vi) **w/o subgraph emb**: remove subgraph embedding $\mathbf{h}_{\mathcal{S}_{h,t}}$ in equation (12).

Supplementary Fig. 1 plots the results. As shown, KnowDDI defeats all these variants. Both"w/o relation emb" and "w/ cosine sim" do not use the relation embedding $\mathbf{h}_r$, which leads to performance drop in comparison to KnowDDI. "w/o relation emb" is slightly better than "w/ cosine sim", which validates the benefit of estimating relevance scores from the datasets. "w/o threshold" suffers from a too dense knowledge subgraph, which has also been observed in Supplementary Fig. 2(d) below. The performance gain of KnowDDI over "w/o learned graph" shows that simply counting on the drug-flow subgraph cannot obtain satisfactory results. "w/o edge update" directly learns a fixed graph structure using generic embeddings which do not encode pair-specific information, while KnowDDI alternatively estimates graph structure and refines both the graph structure and node embeddings to be more predictive of DDI types. "w/o subgraph emb" performs worse, which validates the effectiveness of leveraging subgraph embedding to explicitly encode the local context of the knowledge subgraph.

Supplementary Table 2: A summary of hyperparameters used by KnowDDI.

Hyperparameter	Meaning	Range	DrugBank	TWO-SIDES
lr	learning rate	[0.001,0.005,0.01]	0.005	0.005
lr_decay_rate	decay rate of learning rate	[0.9,0.93,0.95]	0.93	0.93
$weight\_decay\_rate$	weight decay rate in Adam	[5e-6,1e-5,5e-5]	1e-5	1e-5
K	the hop K of neighborhood	[2,3,4]	2	2
emb_dim	dimension of node embedding	[24,32,64]	32	32
rel_emb_dim	dimension of relation embedding	[16,24,32]	32	24
L	number of layers in GraphSAGE	[1,2,3]	2	2
$gcn\_dropout$	dropout rate in GraphSAGE	[0.1,0.2,0.3]	0.2	0.2
T	the times of alternating steps in graph structure learning	[1,2,3]	3	1
P	the length of relational path	[3,4,5]	3	3
MLP_hidden_dim	embedding dimension of MLP in equation (6)	[16,24,32]	16	24
MLP_num_layers	number of MLP layer in equation (6)	[1,2,3]	2	3
$MLP\_dropout$	MLP dropout rate in equation (6)	[0.1,0.2,0.3]	0.2	0.2
γ	the threshold used to screen out the edges	[0.05,0.1,0.15,0.2]	0.05	0.1
α	a hyperparameter to balance the drug-flow subgraph and updated subgraph	[0.3,0.5,0.7]	0.7	0.5

For each hyperparameter, we provide its meaning, range and values chosen on DrugBank and TWOSIDES respectively.

Supplementary Fig. 2: **Sensitivity analysis of KnowDDI on DrugBank.**

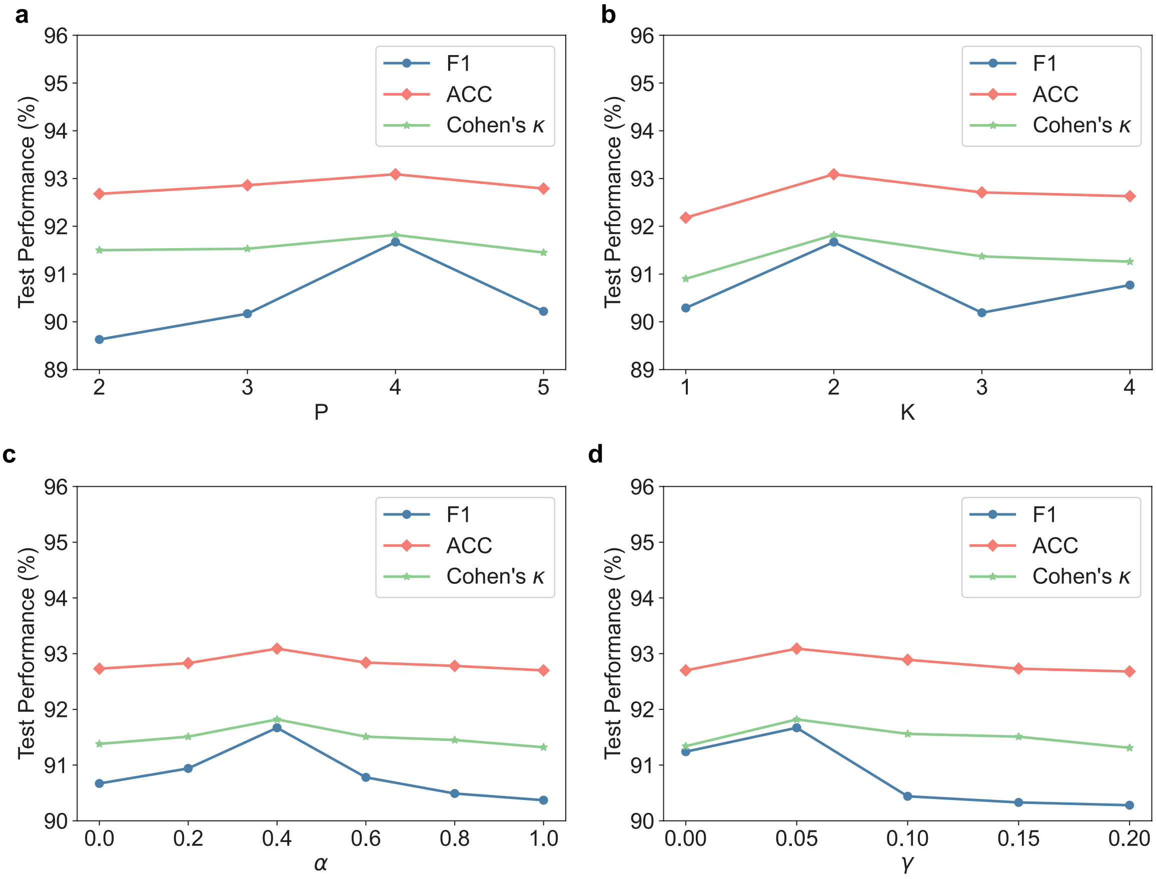

a, Varying the length P of relational path. **b**, Varying the hop K of neighborhood. **c**, Varying α in equation (7). **d**, Varying γ in equation (7). The results are derived from five independent runs (n=5).

Supplementary Note 3: Sensitivity analysis of hyperparameters

Here, we analyze the effect of varying key hyperparameters used in KnowDDI. Supplementary Fig. 2 shows the results. In particular, Supplementary Fig. 2(a) and Supplementary Fig. 2(b) plot the effect of P and K which control the size of drug-flow subgraph and also knowledge subgraph. Larger P and K will result in larger subgraphs. As can be observed, neither too small nor too large a subgraph obtains satisfactory performance. Recall that KnowDDI targets at selectively aggregating global topology information from the combined network and pair-specific information encoded in the local subgraph. A larger subgraph may bring in extra but useless information for the drug-pair. Therefore, one should start with a drug-flow subgraph with proper size by tuning P and K. Supplementary Fig. 2(c) plots the effect of α which balances the contribution of the updated subgraph and the drug-flow subgraph in equation (7). As shown, performance first increases with larger α then decreases, and reaches the best performances at $\alpha = 0.5$. This shows that the learned subgraph can help correct information in the drug-flow subgraph and consequently leads to better performance. Supplementary Fig. 2(d) plots the effect of γ which is the threshold to screen out less informative edges. A larger γ means more edges will be removed and the subgraph becomes more sparse, while a smaller γ keeps a denser graph. As can be observed, γ should be properly selected to only keep those informative edges.

Supplementary Fig. 3: Statistics of relation frequency for three datasets.

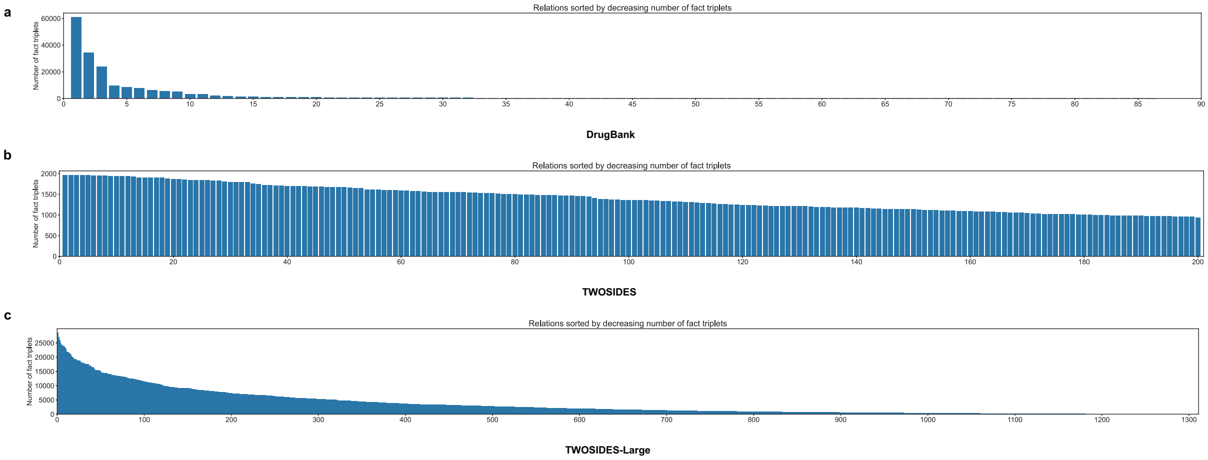

a, DrugBank, **b**, TWOSIDES, and **c**, TWOSIDES-Large. Each bin represents the performance improvement of one relation. From leftside to rightside of x-axis, relations are ordered by descreasing number of associated fact triplets.

Supplementary Fig. 4: **Performance improvement of KnowDDI over SumGNN (the second-best method) on three datasets.**

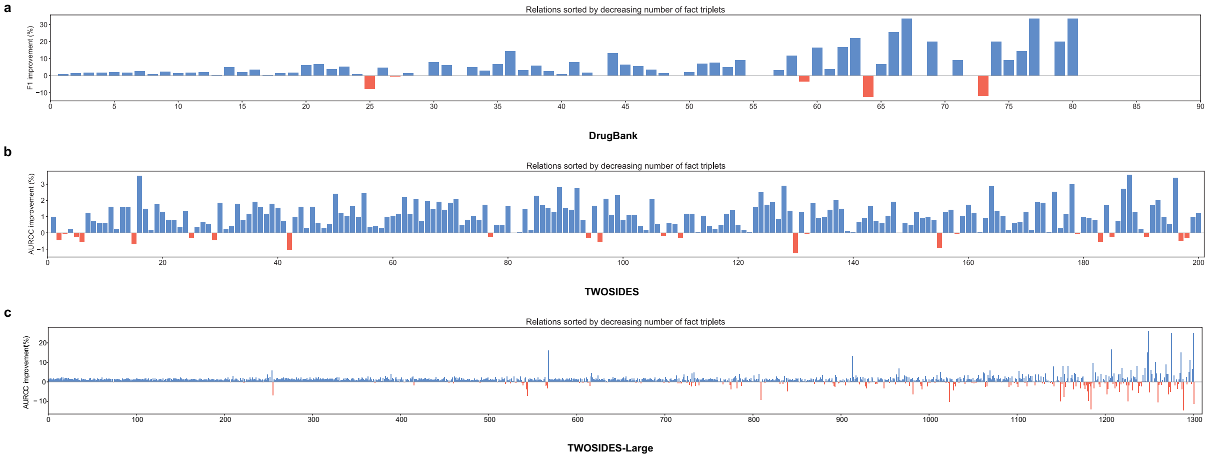

a, DrugBank, **b**, TWOSIDES, and **c**, TWOSIDES-Large. Each bin represents the performance improvement of one relation. From leftside to rightside of x-axis, relations are ordered by descreasing number of associated fact triplets. A higher (lower) bin suggests a larger performance improvement (decrease) for KnowDDI compared to SumGNN. The results are derived from five independent runs (n=5).

Supplementary Table 3: **Performance comparison between KnowDDI and SumGNN on the representative ADRs from the complete TWOSIDES dataset.**

Adverse Drug Reaction	Acute Liver Failure	Acute Myocardial Infarction	Acute Renal Failure	Upper GI Bleeding
KnowDDI	92.23	93.15	90.74	94.78
SumGNN	90.83	91.95	88.90	92.87
Improvement	1.40	1.20	1.84	1.91

The metric is AUROC (%).

Supplementary Note 4: Relation-wise performance improvement

Here, we first report the relation frequency. Supplementary Fig. 3(a) and Supplementary Fig. 3(b) show statistics of relation frequency for DrugBank and TWOSIDES. As can be seen, relations in DrugBank follow long-tail distribution, while relations in TWOSIDES are associated with more comparable fact triples. Recall that TWOSIDES used in this study contains 200 commonly occurring relations with more than 900 fact triplets following SumGNN [23]. This explains why it is easier to predict DDI on TWOSIDES, as can be seen in Table 1. To validate the general effectiveness of KnowDDI, we use the original TWOSIDES [29] which contains 4649441 fact triplets for 645 drugs and 1317 relations, and remove relations with less than 3 fact triplets. Afterwards, we obtain a new dataset denoted as TWOSIDES-Large, which contains 4649430 fact triplets for 645 drugs and 1311 relations. We preprocess it as described in Data preprocessing section. Supplementary Fig. 3(c) shows statistics of relation frequency for TWOSIDES-Large. As shown, relations in TWOSIDES-Large contain varying number of fact triplets.

Next, we provide relation-wise performance improvement of our KnowDDI over SumGNN which obtains the second-best performance in Table 1. Supplementary Fig. 4 shows the results obtained on DrugBank, TWOSIDES, and TWOSIDES-Large. As can be seen, KnowDDI obtains better performance than SumGNN on most relations, including those rare relations. As expected, KnowDDI demonstrates a larger performance gain over SumGNN on TWOSIDES-Large, which contains both sample-sufficient and rare relations, compared to TWOSIDES, which is more balanced in its composition.

Finally, Supplementary Table 3 shows the performance of KnowDDI and the second-best method SumGNN obtained on some important and commonly studied adverse drug reactions (ADRs) which exist in TWOSIDES-Large. Results show that KnowDDI consistently performs better on these relations.

In conclusion, KnowDDI not only excels in terms of overall metrics but also adeptly manages most relations, including those commonly studied. With our open-source approach, we anticipate that KnowDDI can contribute to predicting DDIs for other intriguing relations as well.

Supplementary Fig. 5: **Enlarged enclosing subgraphs plotted in Fig. 3.**

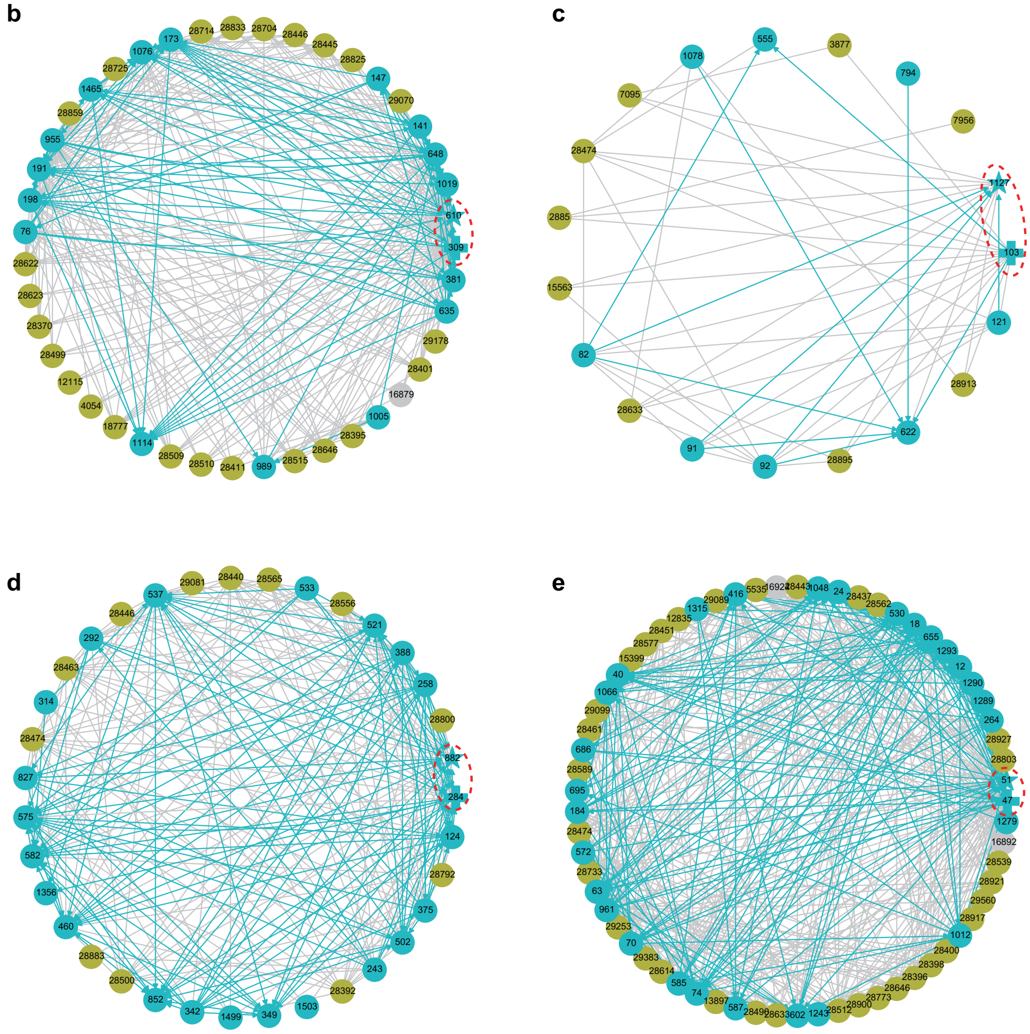

b-e, Enlarged version of enclosing subgraphs plotted in the first column of Fig. 3(b-e).

Supplementary Fig. 6: Case study on resembling drugs.

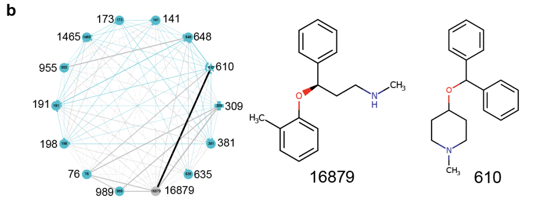

Diphenylpyraline (16879) is an antihistamine used for the treatment of allergic rhinitis, hay fever, and allergic skin disorders.

Atomoxetine (610) is a selective norepinephrine reuptake inhibitor (SNRI) used in the management of Attention Deficit Hyperactivity Disorder (ADHD).

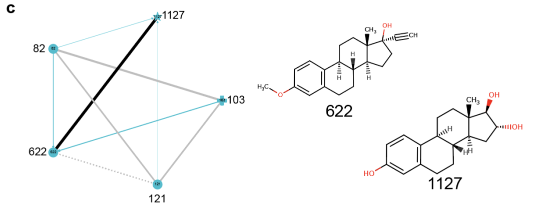

Mestranol (622) is a synthetic estradiol found in oral contraceptive pills for contraception and the treatment of other conditions in the female reproductive system, such as dysmenorrhea and dysfunctional uterine bleeding.

Estriol (1127) is a weak estrogen used to treat vaginal dryness and estrogen deficiency conditions, such as vaginitis and vulvar itching.

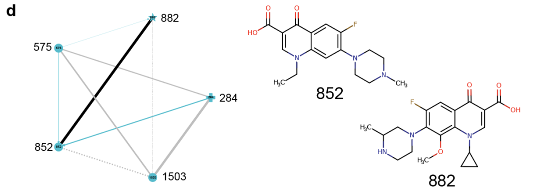

Pefloxacin (852) is an antibiotic used to treat a variety of bacterial infections.

Gatifloxacin (882) is a fourth generation fluoroquinolone used to treat a wide variety of infections in the body.

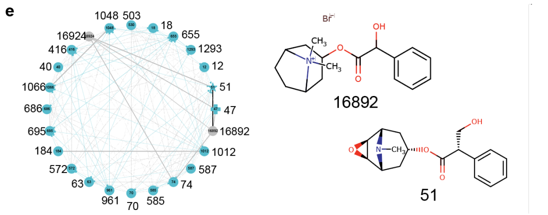

Homatropine (16892) methylbromide is a muscarinic receptor antagonist used to treat peptic ulcers, gastric ulcers, and duodenal ulcers.

Scopolamine (51) is a belladonna alkaloid with anticholinergic effects indicated for the treatment of nausea and vomiting associated with motion sickness and postoperative nausea and vomiting (PONV).

b-e, The molecular graphs and drug efficacy of the node-pair with the largest connection strength in four knowledge subgraphs in Fig. 3(b-e).
