# Supplementary material for: Accurate and interpretable drug-drug interaction prediction enabled by knowledge subgraph learning
Source: Commun Med (Lond). 2024 Mar 28;4:59. doi: 10.1038/s43856-024-00486-y (PMC10978847; doi:10.1038/s43856-024-00486-y)
Supplement: Supplementary file 3 — Description of Additional Supplementary Files [file 43856_2024_486_MOESM3_ESM.pdf]

Description of Additional Supplementary Files

File name: Supplementary Data 1
Description: This file contains all the source data of Fig. 2-4
