# Supplementary material for: Accurate and interpretable drug-drug interaction prediction enabled by knowledge subgraph learning
Source: Commun Med (Lond). 2024 Mar 28;4:59. doi: 10.1038/s43856-024-00486-y (PMC10978847; doi:10.1038/s43856-024-00486-y)
Supplement: Supplementary file 5 — Reporting Summary [file 43856_2024_486_MOESM5_ESM.pdf]

nature portfolio

Corresponding author(s): Quanming Yao

Last updated by author(s): Feb 22, 2024

Reporting Summary

Nature Portfolio wishes to improve the reproducibility of the work that we publish. This form provides structure for consistency and transparency in reporting. For further information on Nature Portfolio policies, see our Editorial Policies and the Editorial Policy Checklist.

Statistics

For all statistical analyses, confirm that the following items are present in the figure legend, table legend, main text, or Methods section.

n/a	Confirmed	
☐	☒	The exact sample size (n) for each experimental group/condition, given as a discrete number and unit of measurement
☐	☒	A statement on whether measurements were taken from distinct samples or whether the same sample was measured repeatedly
☐	☒	The statistical test(s) used AND whether they are one- or two-sided<br>*Only common tests should be described solely by name; describe more complex techniques in the Methods section.*
☐	☒	A description of all covariates tested
☒	☐	A description of any assumptions or corrections, such as tests of normality and adjustment for multiple comparisons
☐	☒	A full description of the statistical parameters including central tendency (e.g. means) or other basic estimates (e.g. regression coefficient) AND variation (e.g. standard deviation) or associated estimates of uncertainty (e.g. confidence intervals)
☒	☐	For null hypothesis testing, the test statistic (e.g. F, t, r) with confidence intervals, effect sizes, degrees of freedom and P value noted<br>*Give P values as exact values whenever suitable.*
☒	☐	For Bayesian analysis, information on the choice of priors and Markov chain Monte Carlo settings
☒	☐	For hierarchical and complex designs, identification of the appropriate level for tests and full reporting of outcomes
☒	☐	Estimates of effect sizes (e.g. Cohen's d, Pearson's r), indicating how they were calculated

Our web collection on statistics for biologists contains articles on many of the points above.

Software and code

Policy information about availability of computer code

Data collection	The processed data analyzed in this paper is available in GitHub repository at https://github.com/LARS-research/KnowDDI/tree/main/data.
Data analysis	We propose a custom algorithm called KnowDDI to handle DDI prediction in this paper. The code implementing KnowDDI is deposited in public available GitHub repository at https://github.com/LARS-research/KnowDDI. The version for this publication is provided in Zenodo with the identifier https://doi.org/10.5281/zenodo.10285646.

For manuscripts utilizing custom algorithms or software that are central to the research but not yet described in published literature, software must be made available to editors and reviewers. We strongly encourage code deposition in a community repository (e.g. GitHub). See the Nature Portfolio guidelines for submitting code & software for further information.

Data

Policy information about availability of data

All manuscripts must include a data availability statement. This statement should provide the following information, where applicable:

- Accession codes, unique identifiers, or web links for publicly available datasets
- A description of any restrictions on data availability
- For clinical datasets or third party data, please ensure that the statement adheres to our policy

All data used in this study are available in supplementary data and public repositories.
For the benchmark DDI datasets, DrugBank dataset can be downloaded from https://bitbucket.org/kaistsystemsbiology/deepddi/src/master/data/.

TWOSIDES dataset can be downloaded from https://tatonettilab.org/resources/nsides/.
Hetionet is obtained from https://het.io.
The processed data analyzed in this paper is available in GitHub repository at https://github.com/LARS-research/KnowDDI/tree/main/data.

Human research participants

Policy information about studies involving human research participants and Sex and Gender in Research.

Reporting on sex and gender	n/a
Population characteristics	n/a
Recruitment	n/a
Ethics oversight	n/a

Note that full information on the approval of the study protocol must also be provided in the manuscript.

Field-specific reporting

Please select the one below that is the best fit for your research. If you are not sure, read the appropriate sections before making your selection.

☒ Life sciences ☐ Behavioural & social sciences ☐ Ecological, evolutionary & environmental sciences

For a reference copy of the document with all sections, see nature.com/documents/nr-reporting-summary-flat.pdf

Life sciences study design

All studies must disclose on these points even when the disclosure is negative.

Sample size	Both DrugBank and TWOSIDES are benchmark datasets and are adopted in previous studies. We follow existing works and do not change the samples size.
Data exclusions	For DrugBank, we remove 0.2% edges which have more than one relation type.<br>For TWOSIDES, we only keep 200 commonly-occurring DDI relation types ranging from Top 600 to Top 800 such that every relation type has at least 900 known drug-drug interaction records.
Replication	We use benchmark datasets and provide the implementation of the proposed KnowDDI method. We also record the random seed which is used to initialize the model parameters. All these measures make replication possible and easy.
Randomization	The samples are split into training, validation and test data following SumGNN (references [22] in the paper). There is no randomization.
Blinding	n/a

Reporting for specific materials, systems and methods

We require information from authors about some types of materials, experimental systems and methods used in many studies. Here, indicate whether each material, system or method listed is relevant to your study. If you are not sure if a list item applies to your research, read the appropriate section before selecting a response.

Materials & experimental systems

n/a	Involved in the study	
☒	☐	Antibodies
☒	☐	Eukaryotic cell lines
☒	☐	Palaeontology and archaeology
☒	☐	Animals and other organisms
☒	☐	Clinical data
☒	☐	Dual use research of concern

Methods

n/a	Involved in the study	
☒	☐	ChIP-seq
☒	☐	Flow cytometry
☒	☐	MRI-based neuroimaging
